# Key Elements and Theoretical Foundations for the Design and Delivery of Text Messages to Boost Medication Adherence in Patients With Diabetes, Hypertension, and Hyperlipidemia: Scoping Review

**DOI:** 10.2196/71982

**Published:** 2025-07-21

**Authors:** Yu-Meng Yang, Tzu Wang, Hsun-Yu Chan, Yen-Ming Huang

**Affiliations:** 1 College of Medicine Graduate Institute of Clinical Pharmacy National Taiwan University Taipei City Taiwan; 2 College of Medicine School of Pharmacy National Taiwan University Taipei City Taiwan; 3 Department of Industrial Education National Taiwan Normal University Taipei City Taiwan; 4 Department of Pharmacy National Taiwan University Hospital Taipei City Taiwan

**Keywords:** adherence, behavioral, design, delivery, medication, message, text, theory

## Abstract

**Background:**

Medication nonadherence in cardiometabolic syndrome negatively impacts patients’ quality of life, health care systems, and economic stability. Despite extensive research, no universally recognized strategy has been established to improve adherence. SMS text messaging has emerged as a widely accessible and cost-effective intervention, particularly when the intervention is structured using behavioral theories.

**Objective:**

This study aimed to review existing literature and identify key factors in the design of SMS text messaging interventions for improving medication adherence among patients with diabetes, hypertension, or hyperlipidemia.

**Methods:**

We conducted a scoping review following the SPIDER (sample, phenomenon of interest, design, evaluation, research type) framework and guided by the PRISMA-ScR (Preferred Reporting Items for Systematic Reviews and Meta-Analyses Extension for Scoping Reviews) checklist. Relevant literature on SMS text messaging interventions was searched in the PubMed and Scopus databases from inception to October 2024. The search terms included “diabetes,” “hypertension,” “hyperlipidemia,” “message,” “text,” “text message,” “app,” “application,” “digital,” “device,” “mobile,” “medical adherence,” and “medication adherence,” combined using logical operators “OR” and “AND.” Full-text articles were analyzed for study design, author, country, year of publication, disease focus, behavioral theory, and the constructs or domains of text messages.

**Results:**

A total of 52 studies investigating SMS text messaging interventions to enhance medication adherence were identified. The targeted conditions included diabetes (26/52, 50%), hypertension (16/52, 31%), and various other chronic diseases (10/52, 19%). More than half of the studies (33/52, 64%) incorporated behavioral theories or techniques in their intervention design, using 19 distinct behavioral models. The most frequently used frameworks were the behavior change technique taxonomy (16/52, 31%) and the capability, opportunity, motivation, and behavior model (6/52, 12%). In addition, 33 (64%) studies implemented tailored messaging strategies, with the most common approach being content customization based on individual patient information (21/52, 40%), followed by personalized timing and frequency of messages (14/52, 27%), to enhance interventions’ adaptability and relevance to users’ needs.

**Conclusions:**

This review highlights critical factors influencing the design of SMS text messaging interventions for medication adherence in the management of diabetes, hypertension, and hyperlipidemia. The findings underscore the importance of integrating behavioral theories and tailoring strategies to optimize patient engagement and intervention effectiveness. Further research is needed to evaluate the impact of different tailoring approaches and translate these insights into practical interventions.

## Introduction

Chronic diseases are a major global health burden [1], profoundly affecting quality of life, health care systems, and economic stability [2]. Hypertension, hyperlipidemia, and diabetes, commonly grouped under cardiometabolic syndrome, are considered leading chronic diseases and are closely linked to a variety of serious health complications. Managing these conditions through lifestyle changes and medication use is essential in reducing their long-term impact on health [2]. The prevalence of chronic diseases has escalated over the past decade, with noncommunicable diseases accounting for 67% of global deaths in 2010, rising to 74% by 2019 [1]. This trend is expected to impose an economic loss of approximately US $47 trillion worldwide by 2030 [3]. Effective management of chronic conditions relies heavily on medication adherence [4], defined as the extent to which individuals follow prescribed medication regimens as suggested by health care professionals [5,6]. High adherence rates are associated with improved clinical outcomes, reduced complications, and lower health care costs [7,8]. However, medication adherence remains suboptimal worldwide, with an estimated global adherence rate of only 50% [9]. This suboptimal adherence highlights the urgent need for effective and scalable strategies to improve adherence, particularly among patients with chronic diseases [10].

A variety of interventions have been implemented to improve medication adherence, including patient education, medication regimen management, pharmacist-led programs, cognitive behavioral therapies, medication reminders, and incentive-based measures [11]. While these approaches have demonstrated efficacy, they are often complex, resource-intensive, and difficult to scale [12-14]. For instance, pharmacist-driven initiatives require significant personnel and financial investments, thus limiting their feasibility in resource-constrained settings [11]. In addition, many existing adherence interventions demand frequent in-person interactions, which can be burdensome for patients and health care providers alike, especially in remote areas or those with mobility challenges [15]. A critical limitation of many adherence interventions is their lack of a solid theoretical foundation, leading to inconsistencies in their effectiveness and applicability across diverse populations [16]. Consequently, there is a growing need for cost-effective, scalable, and theory-driven interventions that can be delivered to reach a broader population [12,14,17].

The rapid proliferation of mobile phone use presents an opportunity to address these challenges [18]. With the widespread adoption of mobile technologies, mobile phone–based interventions present a promising and accessible solution for improving medication adherence [14,19]. As of 2024, approximately 4.3 billion people globally own smartphones [20], yet even those with limited technological literacy or lower socioeconomic status frequently use basic mobile phones [21]. SMS text messaging offers a widely accessible and cost-effective approach that does not require internet connectivity or smartphone technology [22]. Owing to its universal reach, simplicity, and potential for high patient engagement, SMS text messaging–based interventions are particularly well-suited for improving medication adherence, particularly in resource-limited settings [23,24]. The minimal technical demands of SMS text messaging make it particularly appropriate for older adults or individuals with limited digital proficiency [25]. Moreover, SMS text messaging interventions have shown considerable potential in enhancing medication adherence and clinical outcomes while remaining cost-effective. These SMS text messaging–based interventions deliver substantial health benefits while being affordable for both patients and health care systems [26].

Research consistently supports the effectiveness of SMS text messaging–based interventions in enhancing medication adherence, with randomized controlled trials reporting significant improvements in adherence rates [14,22,23,26-28]. Thakkar et al [14] reviewed 16 trials and found that SMS text messaging nearly doubled adherence rates among adults with chronic diseases. Similarly, the study conducted by Burn et al [26] demonstrated that SMS text messaging could reduce cardiovascular events and generate substantial cost savings. Despite these encouraging findings, many studies often overlook the specific content and theoretical grounding of the text messages, which may limit their long-term effectiveness [14]. Furthermore, a significant number of SMS text messaging–based programs either lack a strong theoretical foundation or apply behavioral theories ineffectively [29]. This raises questions about whether theory-driven interventions yield superior outcomes compared with those without a structured theoretical approach [29,30]. In addition, research on SMS text messaging has provided limited insights into their effectiveness across diverse populations and varying patient conditions [27]. The comparative benefits of tailored versus standardized messages remain uncertain, and few reviews have systematically analyzed the personalization strategies used in these interventions [31]. These gaps underscore the need for a comprehensive evaluation of the theoretical models and message-tailoring strategies used in text-based adherence interventions [32].

Given the increasing burden of diabetes, hypertension, and hyperlipidemia and the growing reliance on mobile health interventions, this study aimed to systematically review the literature on SMS text messaging interventions for medication adherence. Specifically, this review synthesized the theoretical frameworks that informed these interventions and investigated the methods used to tailor messages to individual patient needs. By addressing these gaps, this study aimed to contribute to the development of more effective and evidence-based strategies for enhancing medication adherence through mobile health interventions.

## Methods

### Study Design

We used the SPIDER (sample, phenomenon of interest, design, evaluation, research type) framework to develop our search strategy [33], guided by the research question, What are the key components of text messages in medication adherence intervention using digital devices? This study involved formulating a search strategy, applying predefined inclusion criteria for article screening, comparing and selecting relevant studies, and extracting key data. To ensure comprehensive and transparent reporting, this scoping review was conducted in accordance with the PRISMA-ScR (Preferred Reporting Items for Systematic Reviews and Meta-Analyses Extension for Scoping Reviews) guidelines, including the development and implementation of the search strategy [34].

### Search Strategy

We conducted a literature search in 2 electronic databases, PubMed and Scopus, to identify studies that contained text messages delivered via digital tools to enhance medication adherence. The SPIDER framework was applied to structure the research question and guide the search strategy (Multimedia Appendix 1). The key search terms included “diabetes,” “hypertension,” “hyperlipidemia,” “message,” “text,” “text message,” “app,” “application,” “digital,” “device,” “mobile,” “medical adherence,” and “medication adherence.” These terms were combined using the logical operators “OR” and “AND” and were restricted to searches within titles and abstracts only (Multimedia Appendix 2). We reviewed articles published in English from inception to October 2024, excluding those with “review” in the title.

### Selection Process

The first 2 authors (YMY and TW) independently screened titles and abstracts from PubMed and Scopus databases and used EndNote to identify and remove duplicates. Following the screening process, they selected articles based on the predefined inclusion and exclusion criteria (Textbox 1). Studies were excluded if they lacked a clear rationale for message design, failed to describe the message content, or did not align text messages with the corresponding theoretical frameworks. Any disagreements among the reviewers were resolved through discussion, with a third reviewer (YMH) consulted when necessary to reach a consensus. In addition, gray literature was hand-searched using Google Scholar to identify further relevant studies during the full-text review process.

**Inclusion and exclusion criteria of the selected studies.**

**Inclusion criteria**

- Focusing on cardiometabolic syndromes, including diabetes, hypertension, and hyperlipidemia
- Using text messages to assist patients in self-management and improve patients’ medication adherence
- Providing descriptions of the content of text messages used in the study
- Using an experimental design

**Exclusion criteria**

- A review article rather than primary research
- Publications in languages other than English
- Insufficient detailed information about the content of text messages used in the intervention

### Data Extraction and Synthesis

Two reviewers (YMY and TW) extracted data from the full-text articles, including study design, author, year of publication, disease focus, behavioral theory, and the constructs or domains of messages. In addition, detailed information on text messages was collected, such as sending frequency and the content of tailored messages. These text message characteristics across the studies were then evaluated and compared in our review. Frequency tabulation was used to numerically summarize key variables, including disease focus, study design, behavioral theories applied, message frequency, and features of tailored messages.

## Results

### Overview

**Figure 1:**
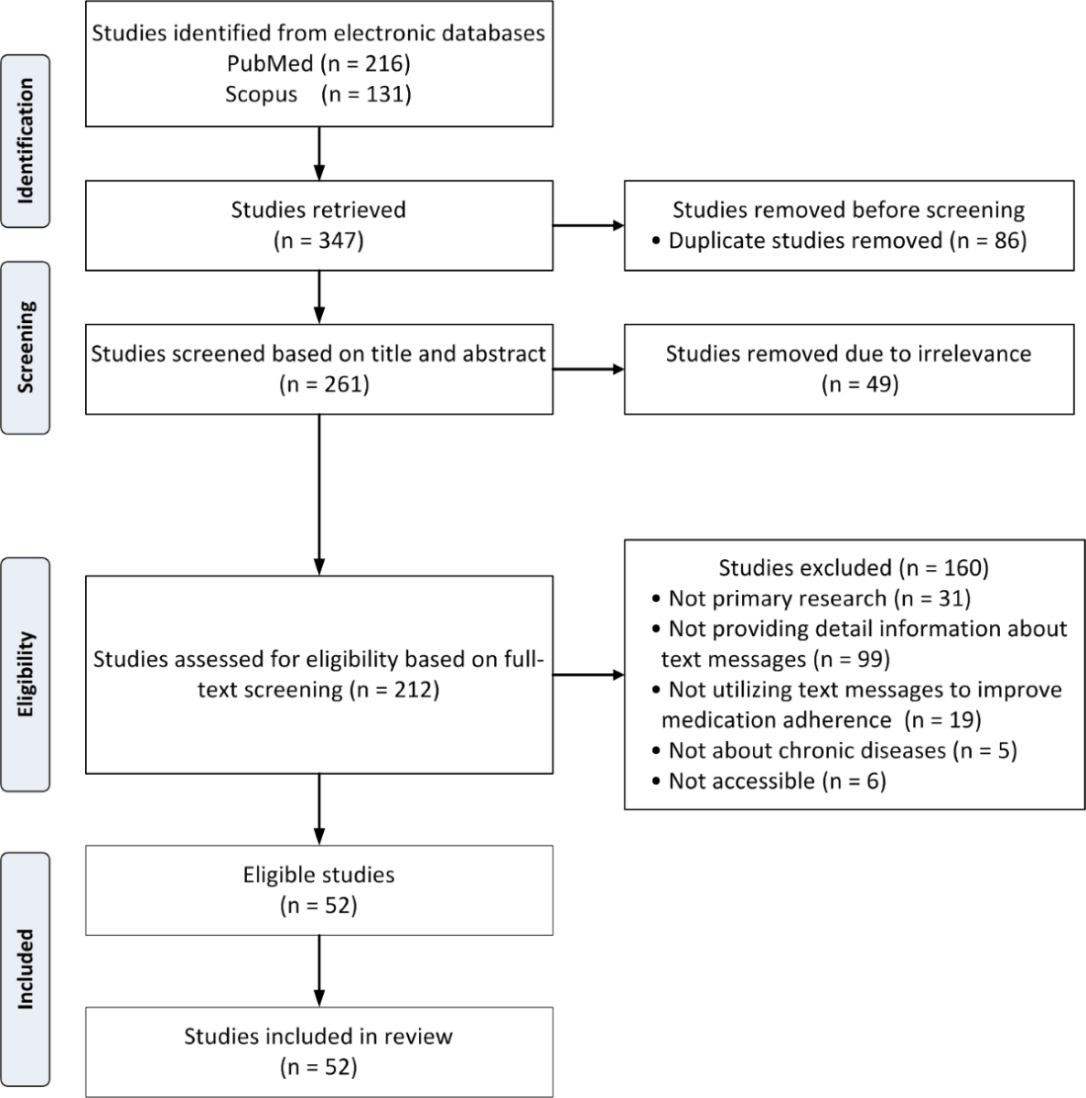
Flow diagram of the literature searching process.

Initially, we identified 216 articles from PubMed and 131 from Scopus; we removed 86 (24.8%) duplicates, leaving 261 (75.2%) studies. After screening titles and abstracts, 49 (14.1%) articles were excluded because of irrelevance. The remaining 212 (61.1%) articles underwent full-text review for eligibility assessment. Of these, 160 (46.1%) studies were excluded for various reasons, including 31 (8.9%) studies not being primary research, 99 (28.5%) lacking detailed information on text messages, 19 (5.5%) not using text messages to improve medication adherence, 5 (1.4%) not focusing on chronic disease, and 6 (1.7%) being inaccessible. Most of the studies excluded because of lack of detail on text messages either failed to explain how the messages were designed, did not connect the messages to any theoretical framework, or referenced theory without showing how it was used to shape the messages. Without this information, we were unable to explore how the messages were tailored or how they related to the underlying theoretical frameworks. Ultimately, 52 (15%) studies [22,35-85] met the inclusion criteria and were included in the review (Figure 1).

### Characteristics of the Included Studies

Among the 52 eligible studies, most focused on a single disease (45/52, 87%). Diabetes (26/52, 50%) was the most commonly studied condition [22,35-37,39,40,44,51,52,56,58,59,63, 65-67,70-74,76,79,80,84,85], including type 1 diabetes (1/52, 2%) [35], type 2 diabetes (22/52, 42%) [22,36,37,40,44,51,52,56,59,63,66,67,70-74,76,79,80,84,85], unspecified diabetes (2/52, 4%) [38,64], and both type 1 and type 2 diabetes (1/52, 2%) [58]. Other conditions investigated included hypertension (16/52, 31%) [38,41-43,45,47,48,55,60-62,68,69,75,81,82], coronary heart disease (1/52, 2%) [57], cardiovascular disease (1/52, 2%) [53], overweight (1/52, 2%) [64], and recurrent stroke prevention (1/52, 2%) [83]. In addition, some studies included participants with multiple or different conditions, such as coronary heart disease and type 2 diabetes (1/52, 2%) [54], diabetes and coronary heart disease (1/52, 2%) [46], hypertension and bipolar disorder (1/52, 2%) [78], type 2 diabetes and hypertension (1/52, 2%) [50], and any chronic disease (2/52, 4%) [49,77] (Table 1).

**Table 1:** Summary and comparison of selected studies on text messages for medication adherence.

Study	Disease focus	Design	Theory	Frequency of messages	Tailored messaging
Mulvaney et al [35], 2012	T1D^a^	Pilot RCT^b^	N^c^	8 to 12 messages per week (mean 10 messages)	Y^d^
Osborn and Mulvaney [36], 2013	T2D^e^	Development and feasibility study	N	2 messages per day	Y
Arora et al [37], 2014	T2D	RCT	N	2 messages per day	N
Bobrow et al [38], 2014	HTN^f^	Pragmatic RCT	N	1 message per week Additional situation-specific message	Y
Gatwood et al [39], 2014	DM^g^	Development and feasibility study	Y	1 message per day	Y
Islam et al [40], 2014	T2D	RCT	Y	1 message per day	N
Buis et al [41], 2015	HTN	RCT	Y	2 messages and 1 assessment per week Self-decided number and timing of daily reminder	Y
Bobrow et al [42], 2016	HTN	Pragmatic RCT	Y	1 message per week Additional situation-specific message	Y
Farris et al [43], 2016	HTN	Cross-sectional	N	—^h^	N
Nelson et al [22], 2016	T2D	Cross-sectional	Y	2 messages per day 1 feedback message per week	Y
Abaza and Marschollek [44], 2017	T2D	Pilot RCT	N	1 message per day	N
Haramiova et al [45], 2017	HTN	Pragmatic RCT	N	Self-decided number and timing of daily reminder messages	Y
Huo et al [46], 2017	DM and CHD^i^	RCT	Y	6 messages per week (from Tuesday to Sunday)	Y
Varleta et al [47], 2017	HTN	RCT	Y	1 message every 12 days (SD 2 d)	N
Bobrow et al [48], 2018	HTN	RCT	Y	1 message per week	N
Prayaga et al [49], 2018	Chronic disease	RCT	N	2 messages per week (on Wednesday and Thursday) Additional situation-specific message	Y
Angellotti et al [50], 2019	T2D and HTN	Single-arm trials	N	Determined by the user	Y
Bartlett et al [51], 2019	T2D	Cross-sectional	Y	—	N
Farmer et al [52], 2019	T2D	Randomized feasibility trial protocol	Y	Up to 4 messages per week (mean 3 messages)	Y
Gong et al [53], 2019	CVD^j^	Pilot study of a cluster-randomized controlled trial	Y	Text message (at 3 PM every 2 d); voice message (at 7 PM every 2 d)	N
Huo et al [54], 2019	CHD and T2D	RCT	Y	6 messages per week (from Tuesday to Sunday, randomly sent at 9 AM, 12 AM, or 4 PM)	Y
Kassavou et al [55], 2019	HTN	Cross-sectional	Y	Determined by the user (1-4 messages per d)	Y
Waller et al [56], 2019	T2D	RCT	Y	For months 1 to 3, 1 message per day; for months 4 to 6; 4 messages per week	Y
Zheng et al [57], 2019	CHD	RCT	Y	6 messages per week (from Tuesday to Sunday)	Y
Adu et al [58], 2020	T1D and T2D	Development and feasibility study	Y	—	Y
Bartlett et al [59], 2020	T2D	Message development and fidelity study	Y	—	N
Bhandari et al [60], 2020	HTN	RCT	Y	3 messages per week	N
Champoux et al [61], 2020	HTN	Development and feasibility study	Y	2 messages per week Monthly blood pressure feedback message	Y
Jahan et al [62], 2020	HTN	RCT	N	5 messages in the first month, and then 1 message per week for the following 4 months	N
Kundury and Hathur [63], 2020	T2D	Pilot RCT	N	1 message per week	N
Li et al [64], 2020	Overweight	RCT	Y	3 or 5 per week, depending on the allocation	Y
Owolabi et al [65], 2020	DM	RCT	N	Determined by the user	Y
Sadanshiv et al [66], 2020	T2D	RCT	Y	2 messages per week	Y
Schoenthaler et al [67], 2020	T2D	Cross-sectional	Y	4 messages per day 1 feedback message per week	Y
Zhai et al [68], 2020	HTN	RCT	N	1 message per 3 days	N
Campos et al [69], 2021	HTN	Pilot RCT	N	Once per week (multiple messages)	Y
Farmer et al [70], 2021	T2D	RCT	Y	3 to 4 times weekly	Y
Lauffenburger et al [71], 2021	T2D	Pragmatic RCT	N	—	N
Leon et al [72], 2021	T2D	RCT	Y	Up to 4 times per week	Y
Sharp et al [73], 2021	T2D	RCT	N	Up to 7 messages per week	N
Bartlett et al [74], 2022	T2D	RCT feasibility	Y	Up to 4 text messages per week	Y
Bhandari et al [75], 2022	HTN	Pilot RCT	Y	3 times per week in the morning (9 AM-10 AM)	Y
Farmer et al [76], 2022	T2D	Protocol of RCT	Y	Up to 4 messages per week	N
Kershaw et al [77], 2022	Nonspecific chronic disease	Pilot RCT	N	—	N
Levin et al [78], 2022	HTN among patients with bipolar	2-stage RCT	Y	High intensity (starts off with 1 reminder per day for 2 months and tapers down to 1 reminder per week for another 2 months) or Low intensity (1 reminder per week for 4 months)	Y
Mandal et al [79], 2022	T2D	RCT	N	Questions on individualized healthy living goals and patients’ diabetes quality of life were sent weekly, and the remaining messages were sent daily	Y
Philis-Tsimikas et al [80], 2022	T2D	RCT	Y	2 to 3 messages per day (frequency tapering)	N
Van Emmenis et al [81], 2022	HTN	Cross-sectional	Y	Not specified	Y
Asgary et al [82], 2023	HTN	Cross-sectional	Y	1 message every other day	N
SPRINT INDIA trial collaborators [83], 2023	Recurrent stroke prevention	RCT	N	Daily text message for the first 6 weeks, then a text message twice a week until 6 months and, thereafter, 1 message a week until 1 year	Y
Zamanillo-Campos et al [84], 2023	T2D	Pragmatic RCT	Y	Up to 5 messages per week (from Monday to Friday)	Y
Zamanillo-Campos et al [85], 2024	T2D	RCT	Y	Up to 5 SMS text messages per week	Y

^a^T1D: type 1 diabetes.

^b^RCT: randomized controlled trial.

^c^N: did not use theory or tailored messaging on text messages for medication adherence.

^d^Y: used theory or tailored messaging on text messages for medication adherence.

^e^T2D: type 2 diabetes.

^f^HTN: hypertension.

^g^DM: diabetes mellitus.

^h^Not available.

^i^CHD: coronary heart disease.

^j^CVD: cardiovascular disease.

Regarding study design, 39 (75%) studies used a randomized controlled trial approach [35,37,38,40-42,44-49,52-54,56,57,60,62-66,68-80,83-85], 7 (14%) were cross-sectional studies [22,43,51,55,67,81,82], and 5 (10%) focused on message development and feasibility [36,39,58,59,61] (Table 1). Furthermore, 33 (64%) studies incorporated behavioral theories or techniques [22,39-42,46-48,51-61,64,66,67,70,72,74-76,78,80-82,84,85], while an equal number (33/52, 64%) incorporated tailored message design [22,35,36,38,39,41, 42,45,46,49,50,52,54-58,61,64-67,69,70,72,74,75,78,79,81,83-85] (Table 1).

### Features of the Behavioral Theory or Technique

Over half of the included studies incorporated behavioral theories or techniques (33/52, 64%) [22,39-42,46-48,51-61,64,66,67,70,72,74-76,78,80-82,84,85]. Among these, 19 distinct behavioral theories or techniques were used during message development (Table 2). The most frequently applied framework was the behavior change technique taxonomy (16/52, 31%) [42,48,51-57,59,60,72,74-76,84], followed by the capability, motivation, opportunity, behavior (COM-B) model (6/52, 12%) [56,60,67,70,72,75], the transtheoretical model of behavioral change (4/52, 8%) [40,53,66,80], and the social cognitive theory (4/52, 8%) [47,61,80,82]. Among the 33 studies incorporating theoretical frameworks or techniques in message design, 19 (37%) used a single theory or technique [22,42,46,47,51,52,54,55,57,58,61,66,70,74,76,78,81,84,85], while 14 (27%) applied multiple theories or techniques [39-41,48,53,56,59,60,64,67,72,75,80,82]. Consequently, the total number of theories or techniques used exceeded the number of studies.

**Table 2:** Theories and domains applied in the studies.

Study	Theory	Construct or domain of messages
Mulvaney et al [35], 2012	—^a^	Total number of messages: 595 Domains: 9 Burnout and stress, sports and exercise, communication, social support and stigma, social situations, time pressure and planning, reminders, carbohydrates, and autonomy support
Osborn and Mulvaney [36], 2013	—	Total number of messages: — Domains: 17 Barriers to accessing medications (10-13 items), believing medications are not important (10-13 items), believing medications are harmful (10-13 items), complexity of regimen (10-13 items), cost of medications (10-13 items), decision to omit doses (10-13 items), experiencing side effects (10-13 items), fear of dependence (10-13 items), fear of side effects (10-13 items), feeling stigmatized (10-13 items), forgetfulness (10-13 items), lack of belief in the benefits of medications (10-13 items), lack of information about medications, lack of motivation to take medications (10-13 items), lack of social support (10-13 items), lack of diabetes symptoms (10-13 items), and tired of taking medications (10-13 items)
Arora et al [37], 2014	—	Total number of messages: — Domains: 4 Educational or motivational (1 per d), medication reminders (3 per wk), healthy living challenges (2 per wk), and trivia (2 per wk)
Bobrow et al [38], 2014	—	Total number of messages: — Domains: 7 Behavioral change taxonomy (4 domains): goals and planning (2 items), repetition and substitution (3 items), social support (2 items), and natural consequences (1 item) Contextual messages (3 domains, send when appropriate to the context): appointment reminder SMS text messages (1 item), in response to missing a scheduled appointment (1 item), and messages related to specific issues (3 items)
Gatwood et al [39], 2014	Health beliefs model Self-determination theory	Total number of messages: 296 Domains: 7 Severity (low and high), susceptibility (low and high), barriers (low and high), benefits (low and high), competence (low, medium, and high), motivation (low, medium, and high), and regulation (low, medium, and high)
Islam et al [40], 2014	Behavioral learning theory Transtheoretical model of behavioral change	Total number of messages: 90 Domains: —
Buis et al [41], 2015	Theory of self-regulation framework Health belief model	Total number of messages: — Domains: 3 Medication reminder, educational message, and satisfaction assessment
Bobrow et al [42], 2016	Behavior change techniques	Total number of messages: — Domains: 16 Repetition and substitution (4 domains): habit formation, behavior substitution, behavioral rehearsal or practice, and generalization of a target behavior Natural consequences (3 domains): health consequences, salience of consequences, and anticipated regret Goals and planning (6 domains): action planning, problem-solving, commitment, goal setting (outcome), behavioral contract, and review of behavior goals Social support (3 domains): practical, general, and emotional
Farris et al [43], 2016	—	Total number of messages: 130 Domains: 5 Disease beliefs (30 items), medication necessity (30 items), medication concerns (30 items), forgetfulness (30 items), and positive reinforcement (10 items)
Nelson et al [22], 2016	Information-motivation-behavioral skills model	Total number of messages: — Domains: 8 Medication adherence (3 per wk), medication regimen-specific (1 per wk), exercise (1 per wk), diet (1 per wk), SMBG^b^ (1 per wk), daily text assessing adherence (1 per d), weekly text providing feedback (1 per wk), and A1C^c^ test (after A1C test)
Abaza and Marschollek [44], 2017	—	Total number of messages: 84 Domains: 7 Diabetes knowledge and effects on social and personal life (sent on Saturdays), diet (sent on Sundays), physical activity (sent on Mondays), smoking, foot care, and diabetes complications (sent on Tuesdays), medications and side effects (sent on Wednesdays), tests and blood sugar measurement (sent on Thursdays), and hyper- and hypoglycemia (sent on Fridays)
Haramiova et al [45], 2017	—	Total number of messages: 1 Domains: 1 SMS text messaging reminder
Huo et al [46], 2017	Information-motivation-behavioral skills model	CHAT^d^ trial Total number of messages: 280 Domains: 5 General education (CVD^e^; 2 per wk), blood pressure control (2 per wk), medication adherence (1 per wk), physical activity (1 per wk), and smoking cessation (1 per wk; only smoking user received) CHAT-DM^f^ trial Total number of messages: 270 Domains: 6 General education (CVD; 1 per wk), blood pressure control (1 per wk), medication adherence (1 per wk), physical activity (1 per wk), 2-way diabetes management (1 per wk), and lifestyle intervention (1 per wk)
Varleta et al [47], 2017	Social cognitive theory	Total number of messages: 15 Domains: —
Bobrow et al [48], 2018	I-Change model Behavior change techniques	Total number of messages: — Domains: 4 Repetition and substitution, natural consequences, goals and planning, and social support
Prayaga et al [49], 2018	—	Total number of messages: 22 Domains: 3 Refill reminders (7 items), actions for missed refills (6 items), and others (9 items)
Angellotti et al [50], 2019	—	Total number of messages: — Domains: 3 Exercise, diet, and medication adherence
Bartlett et al [51], 2019	Behavior change techniques	Total number of messages: — Domains: 15 Goals and planning, feedback and monitoring, social support, shaping knowledge, natural consequences, comparison of behavior, associations presented together with antecedents, repetition and substitution, comparison of outcomes, reward and threat, regulation, identity, self-belief, dealing with side effects, and concerns with health care system and medications
Farmer et al [52], 2019	Behavior change techniques	Total number of messages: — Domains: 3 Medication, diet and exercise, and belief
Gong et al [53], 2019	Health belief model Transtheoretical model Behavior change techniques	Total number of messages: — Domains: 6 Management of metabolic risk factors, medication adherence, tobacco and alcohol control, dietary change, exercise and rehabilitation, and psychological support
Huo et al [54], 2019	Behavioral change techniques	Total number of messages: 270 Domains: 6 General education (CVD; 1 per wk), blood pressure control (1 per wk), medication adherence (1 per wk), physical activity (1 per wk), 2-way diabetes management (1 per wk), and lifestyle intervention (1 per wk)
Kassavou et al [55], 2019	Behavioral change techniques	Total number of messages: — Domains: 3 Patient and public involvement and engagement messages (2 domains): hospital open event and science festival Examples of intentional nonadherence and nonintentional nonadherence tailoring messages
Waller et al [56], 2019	COM-B^g^ model Behavioral change techniques	Total number of messages: 151 Domains: 6 Nutrition (47 items), physical activity (47 items), diabetes care (26 items), weight management (13 items), medication adherence (9 items), and smoking cessation (9 items)
Zheng et al [57], 2019	Behavioral change techniques	Total number of messages: 280 Domains: 5 General education (CVD; 2 per wk), blood pressure control (2 per wk), medication adherence (1 per wk), physical activity (1 per wk), and smoking cessation (1 per wk; only smoking user received)
Adu et al [58], 2020	Information-motivation-behavioral skills model	Total number of messages: — Domains: 7 DM^h^ self-management (6 domains): what is diabetes? health food choices, physical activity, medication use, monitoring blood glucose, and reducing the risk of complications Semipersonalized messages based on input health data
Bartlett et al [59], 2020	Behavioral change techniques Concern and beliefs	Total number of messages: 306 Domains: 34 Content related (3 domains): medication adherence, beliefs and concerns, and diet management Theory related (31 domains): problem-solving, action planning, self-monitoring of behavior, social support (unspecified), social support (practical), social support (emotional), information about antecedents, information about social and environmental consequences, anticipated regret, information about emotional consequences, social comparison, information about others’ approval, prompts or cues, habit formation, credible source, pros and cons, comparative imaginings of future outcomes, social reward, reduce negative emotions, restructuring the physical environment, identity associated with changed behavior, verbal persuasion about capability, mental rehearsal of successful performance, self-talk, difficulties with side effects, difficulties remembering and understanding the medication regimen, beliefs around medication in general and Western medicines specifically, perceived risks of taking medication, beliefs about medication necessity, social influence around taking medications, and health care system–related concerns
Bhandari et al [60], 2020	COM-B model Behavioral change techniques	Total number of messages: 36 to 40 Domains: 4 Literacy of hypertension and its treatment, beliefs about the consequences of diseases or faith in traditional medicine or local herbs, nonadherence or forgetting to take medicine, and resistance to behavior modification or unhealthy habits
Champoux et al [61], 2020	Social cognitive theory	Total number of messages: — Domains: 3 Healthy behavior (1 per wk), blood pressure self-monitoring (1 per wk), facilitated appointment and transportation (1 per mo)
Jahan et al [62], 2020	—	Total number of messages: — Domains: 5 Reduce sodium intake (salt), avoid eating oily and fatty foods, eat more fruits and vegetables, exercise regularly, and take your medicine regularly
Kundury and Hathur [63], 2020	—	Total number of messages: 6 Domains: —
Li et al [64], 2020	Self-determination theory Cognitive behavioral therapy	Total number of messages: — Domains: 6 Medicine, motivation, nutrition, activeness, coping strategies, and weight management
Owolabi et al [65], 2020	—	Total number of messages: — Domains: 5 Core messages, healthy eating, stress and mood management message, exercise, and reminders
Sadanshiv et al [66], 2020	Transtheoretical model	Total number of messages: — Domains: 2 Diet control and exercise
Schoenthaler et al [67], 2020	Technology acceptance model COM-B model	Total number of messages: — Domains: 5 Completion-based (first wk, first mo, and third mo), response-based (1 per wk), activity-based (1 per wk), combination of response-based, and individual
Zhai et al [68], 2020	—	Total number of messages: 30 Domains: 3 Knowledge about hypertension, lifestyle modifications, and measures to improve medication adherence
Campos et al [69], 2021	—	Total number of messages: — Domains: 2 Adherence checkpoint and self-monitoring phase
Farmer et al [70], 2021	COM-B model	Total number of messages: — Domains: 2 Medication and healthy lifestyle and enhancing well-being
Lauffenburger et al [71], 2021	—	Total number of messages: 128 Domains: 5 Framing: classified as neutral, positive, or negative; and observed feedback, whereby the SMS text message included the number of days in the previous week that patients had evidence of medication-taking (ie, 0-7), social reinforcement (ie, mentioning loved ones in the text), the nature of the content, either providing a medication reminder or information about medications or lifestyle, and reflection, where the texts were designed to invoke introspection, such as including a reflective question
Leon et al [72], 2021	COM-B model Behavior change techniques	Total number of messages: 156 diabetes content-related messages and 16 trial-related messages Domains: 4 Content related (2 domains): enhance medical adherence (ie, medication collection, medical appointment, taking the medication regularly, and medical adherence support), and enhance general health and well-being (ie, general health and well-being, exercise, nutrition and health, smoking and drinking, and stress management) Theory related (2 domains): COM-B, and behavior change techniques
Sharp et al [73], 2021	—	Total number of messages: — Domains: 7 Medication reminder, refill reminder, appointment reminder, goal monitoring, glucose monitoring, self-efficacy, and motivation
Bartlett et al [74], 2022	Behavior change techniques	Total number of messages: — Domains: 16 Content related (2 domains): medication adherence based on behavior change techniques and diet and physical activity Theory related (14 domains): action self-efficacy, necessity, concerns, intention, automaticity, maintenance self-efficacy, recovery self-efficacy, action planning, coping planning, action control, prompts and cues, social support, satisfaction with experienced consequences, and risk perception
Bhandari et al [75], 2022	COM-B model Behavior change techniques	Total number of messages: — Domains: 9 Content related (7 domains): literacy, beliefs of disease, faith in herbs, nonadherence, forgetting, dietary, resistance to behavior change, and stigma Theory related (2 domains): COM-B and matched items from the behavior change techniques
Farmer et al [76], 2022	Behavior change techniques	Total number of messages: — Domains: 3 Medication adherence, beliefs and concerns, and diet management
Kershaw et al [77], 2022	—	Total number of messages: 65 Domains: 3 Educational or motivational medication reminder messages (32 items), general health information messages (32 items), and 2-way messages—asking about mood (1 item)
Levin et al [78], 2022	Attitude-social influence-efficacy theoretical model	Total number of messages: — Domains: 12 Hypertension knowledge, bipolar knowledge, benefits of blood pressure medication, benefits of bipolar medication, making peace with medication, social support, self-efficacy, medication routines, spiritual, self-esteem, social comparison, and custom
Mandal et al [79], 2022	—	Total number of messages: — Domains: 6 Sleep quality, physical activities, medication adherence, healthy eating, living goals, and diabetes control
Philis-Tsimikas et al [80], 2022	Operant conditioning Social cognitive theory Social ecologic model Theory of planned behavior Transtheoretical model or stages of change	Total number of messages: — Domains: 5 Clinical Indicators (goals and planning), medication (social support), nutrition (antecedents), physical activity (shaping knowledge), and well-being (regulation)
Van Emmenis et al [81], 2022	Perceptions and practicalities approach	Total number of messages: — Domains: 4 Medication reminder, feedback, information, and support
Asgary et al [82], 2023	Social cognitive theory Self-determination theory	Total number of messages: — Domains: 3 Knowledge (knowledge, barriers, and self-sufficiency), motivation (motivation, importance, and social support), maintenance (goals, self-monitoring, and self-efficacy)
SPRINT INDIA trial collaborators [83], 2023	—	Total number of messages: 68 Domains: 3 Physical activity, blood pressure control, and diabetes control
Zamanillo-Campos et al [84], 2023	Behavior change techniques	Total number of messages: — Domains: 7 Diabetes knowledge, medication, physical activity, healthy diet, prevention of overweight, healthy diet, and reminder
Zamanillo-Campos et al [85], 2024	Behavior change wheel framework	Total number of messages: — Domains: 6 Diabetes knowledge, medication, physical activity, healthy diet, prevention of overweight, and reminder

^a^Not available.

^b^SMBG: self-monitoring of blood glucose.

^c^A1C: glycated hemoglobin.

^d^CHAT: cardiovascular health and text messaging.

^e^CVD: cardiovascular disease.

^f^CHAT-DM: cardiovascular health and text messaging–diabetes mellitus.

^g^COM-B: capability, opportunity, motivation, and behavior.

^h^DM: diabetes mellitus.

### Domain or Constructs

The design of the SMS text messages incorporated between 1 and 34 domains or constructs, although 3 (6%) studies did not specify the exact number used [40,47,63] (Table 2). Across all 52 studies, 8 distinct domain categories were identified: behavioral factors, knowledge and education, social and psychological support, lifestyle and health behaviors, clinical and management goals, barriers to access, cultural and belief systems, and theoretical constructs. Among these, behavioral factors were the most frequently addressed in interventions, followed by lifestyle and health behaviors, as well as knowledge and education. Behavioral factors focused on reducing the burden of medication-taking and encouraging behavior change by incorporating adherence reminders, providing cues or prompts, and fostering positive habits to support consistent medication use. Furthermore, lifestyle-related factors, including diet and nutrition as well as physical activity and exercise, played a significant role in intervention design.

### Tailored Messages

In addition to generic messages sent uniformly to all recipients, 33 (64%) studies incorporated tailored messages customized to individual characteristics or needs [22,35,36,38,39,41,42,45,46,49,50,52,54-58,61,64-67,69, 70,72,74,75,78,79,81,83-85]. As summarized in Table 3, these studies used various methods and strategies to ensure that message content aligned with users’ specific conditions.

**Table 3:** Methods for tailoring message content to match users’ specific conditions.

Study	Tailored method
Mulvaney et al [35], 2012	Timing and frequency Evaluation before the intervention (top 3 barriers in the Diabetes Adherence Barriers Assessment)
Osborn and Mulvaney [36], 2013	Timing and frequency Evaluation before the intervention (top 3 barriers in the Diabetes Medication Knowledge Questionnaire, Medicines for Diabetes Questionnaire, Barriers to Diabetes Adherence measure, and Medication Adherence Self-Efficacy Scale)
Bobrow et al [38], 2014	Individual information (name and scheduled appointment)
Gatwood et al [39], 2014	Timing and frequency Individual information (name, age, and medication regimens) Evaluation before the intervention (patient level [low, medium, or high] categorized by the health beliefs model and self-determination theory)
Buis et al [41], 2015	Timing and frequency Individual information (name)
Bobrow et al [42], 2016	Individual information (name, birthday, and scheduled appointment)
Nelson et al [22], 2016	Individual information (medication names) Evaluation before the intervention (top 4 barriers in the Information-motivation-behavioral skills model evaluation) Evaluation during the intervention (daily survey on medication adherence)
Haramiova et al [45], 2017	Timing and number of daily reminders Individual information (medication names, dosage, and frequency)
Huo et al [46], 2017	Individual information (name and smoking status)
Prayaga et al [49], 2018	Users’ responses (the refill process and their identity)
Angellotti et al [50], 2019	Evaluation before the intervention (behavioral economics, such as limiting choice overload and setting goal gradients, and patient-specified personal health goals identified in the baseline questionnaire)
Farmer et al [52], 2019	Individual information (time since starting new glucose, blood pressure and cholesterol-lowering medication, and smoking status)
Huo et al [54], 2019	Individual information (name and smoking status)
Kassavou et al [55], 2019	Timing and frequency Individual information (name and medication name) Evaluation during the intervention (adjustment questionnaire and prescription plan data, considering the levels of intentional nonadherence and nonintentional nonadherence)
Waller et al [56], 2019	Individual information (name and smoking status)
Zheng et al [57], 2019	Individual information (name and smoking status)
Adu et al [58], 2020	Evaluation during the intervention (self-monitoring blood sugar)
Champoux et al [61], 2020	Evaluation during the intervention (self-monitoring blood pressure)
Li et al [64], 2020	Individual information (name) Other (different greetings according to the time and day that the SMS text message was sent, unique content each time)
Owolabi et al [65], 2020	Timing and frequency Individual information (name)
Sadanshiv et al [66], 2020	Timing and frequency
Schoenthaler et al [67], 2020	Evaluation during the intervention (daily patient-reported outcome assessments) Users’ responses (response rate)
Campos et al [69], 2021	Evaluation during the intervention (adherence checkpoint question message)
Farmer et al [70], 2021	Timing and frequency Individual information (favor language)
Leon et al [72], 2021	Timing and frequency Individual information (favor language)
Bartlett et al [74], 2022	Timing and frequency Users’ responses (users’ preference to “like” or “dislike” the message)
Bhandari et al [75], 2022	Individual information (smoking status and alcohol use status)
Levin et al [78], 2022	Individual information (name)
Mandal et al [79], 2022	Timing and frequency Individual information (favor language) Evaluation before the intervention (healthy living goal)
Van Emmenis et al [81], 2022	Timing and frequency
SPRINT INDIA trial collaborators [83], 2023	Timing and frequency
Zamanillo-Campos et al [84], 2023	Individual information (clinical data such as the presence of other chronic diseases or diabetes-related complications)
Zamanillo-Campos et al [85], 2024	Individual information (clinical data, such as the presence of other chronic diseases or diabetes-related complications)

Among the studies using tailored text messages, the most common approach involved customizing message content based on users’ personal information. This strategy was applied in 21 (40%) studies [22,38,39,41,42,45,46,52,54-57,64,65,70,72,75,78,79,84,85], incorporating details such as users’ names, dates of birth, medication use, dosage and frequency, scheduled follow-up or refill appointments, smoking status, and preferred language. In addition, 14 (27%) studies allowed users to customize message timing and frequency [35,36,39,41,45,55,65,66,70,72,74,79,81,83], providing greater flexibility to meet individual needs. Moreover, 6 (12%) studies tailored messages based on assessments before the intervention [22,35,36,39,50,79]. Of these, 3 (6%) evaluated users’ top 3 or 4 barriers to medication adherence and provided targeted messages to address these challenges [22,35,36]. Two (4%) studies used baseline questionnaires to identify patients’ personal health goals and designed messages accordingly [50,79]. One (2%) study applied the health belief model and self-determination theory to categorize patients into different levels and tailored messages based on these classifications [39].

Furthermore, 6 (12%) studies tailored messages based on ongoing assessments conducted during the intervention [22,55,58,61,67,69]. Among these, 4 (8%) adjusted messages based on users’ daily survey or questionnaire responses [22,55,67,69], while 2 (4%) modified messages according to users’ clinical data, such as self-monitored blood pressure or blood glucose levels [58,61]. In addition, 3 (6%) studies personalized messages in response to users’ feedback [49,67,74], considering factors such as medication refill status, response rates to previous messages, and user preferences for content.

To further illustrate tailored messaging approaches, several representative studies were highlighted. These studies were carefully chosen to showcase a variety of tailoring strategies used across the broader group of included studies, such as providing clear explanations for message design, methods for crafting tailored messages, and incorporating patient feedback into the design and delivery of these messages. Mulvaney et al [35] developed messages addressing adherence barriers identified through the Barriers to Diabetes Adherence instrument. Participants received 75% of their messages tailored to their top 3 adherence barriers, with the remaining 25% randomly selected from a broader message pool. In addition, users could schedule messages at specific times in 15-minute intervals and set delivery frequency to daily, weekly, or weekends only. Gatwood et al [39] designed messages using constructs from the self-determination theory and health belief model. A Likert-type survey categorized users into high, medium, or low levels for each theoretical construct, with participants initially receiving 2 messages tailored to their level, followed by a third message from a higher level (eg, low-low-medium). The REACH program by Nelson et al [22] delivered daily interactive messages assessing medication adherence. If users responded “no,” a follow-up message prompted them to reflect on their reasons for nonadherence. The study conducted by Prayaga et al [49] used mPulse Mobile to deliver interactive messages focused on medication refill adherence. Users received reminders near refill dates and were asked to respond. If they did not reply within 2 hours or provided an irrelevant response, they received an additional reminder. Adu et al [58] developed My Care Hub, which provided semi-individualized feedback based on users’ logged blood glucose levels. If readings were within the clinically recommended range, messages encouraged continued self-management. If levels were outside the normal range, messages offered problem-solving suggestions for managing high or low blood glucose. Finally, Bartlett et al [74] implemented a preference-based tailoring approach. After receiving a message, users could respond with “like” or “dislike.” For medication adherence messages, liking a message doubled the likelihood of receiving future messages based on the same behavior change technique, while disliking a message halved the probability of receiving similar messages in the future.

## Discussion

### Main Findings

This review identified 52 SMS text messaging interventions designed to enhance medication adherence in patients with diabetes, hypertension, and hyperlipidemia. The findings underscore the varied strategies used to tailor these interventions to improve patient engagement and adherence. Consistent with previous research, personalized text messages that incorporate theoretical frameworks and adapt to real-world contexts show promise in supporting medication adherence [86].

The review categorized text messages into 2 main approaches: content-driven and theory-based designs. Content-driven messages focus on delivering specific information to participants, typically addressing goals such as medication adherence, healthy eating, and regular exercise. In contrast, theory-based messages are structured around behavioral theories, shaping their content based on key theoretical constructs. While some studies adopted only one of these approaches, others combined both strategies. For example, Zamanillo-Campos et al [85] demonstrated that their intervention integrated both targeted health objectives and behavior change techniques, effectively bridging theoretical foundations with practical applications in behavioral interventions. By merging the structured guidance of behavioral theories with the clarity of direct messaging, their study illustrates a comprehensive approach to intervention design.

### Behavioral Theory or Technique Use in Interventions

The behavior change technique taxonomy is a comprehensive and standardized framework designed to identify, classify, and describe the active components of behavior change interventions [87]. The taxonomy, consisting of 93 distinct techniques, provides structured approaches for designing and implementing behavioral interventions. In this review, behavior change techniques emerged as the most frequently applied behavioral framework, either used independently or in combination with other models. Behavioral theories and techniques often serve as the foundation for interventions, guiding the development of text message content. For instance, Bartlett et al [59] structured and refined messages based on behavior change techniques through workshops, focus groups, acceptability surveys, and fidelity assessments. Their library of brief messages demonstrated both the applicability and acceptance of interventions based on behavior change techniques.

The COM-B model posits that behavior change requires 3 essential components: capability, opportunity, and motivation [88]. When integrated with the behavior change wheel, COM-B helps identify intervention functions and policy strategies to address behavioral barriers and enablers [88]. Schoenthaler et al [67] applied the COM-B model to text message design, showing that multiple constructs of capability, opportunity, and motivation could be incorporated into message components simultaneously. In addition, they linked their messaging system to patient-reported outcomes, allowing real-time feedback on how patients felt upon receiving messages and informing the development of future behavioral messages.

The transtheoretical model of behavior change conceptualizes behavior change as a dynamic, nonlinear process consisting of 5 stages: precontemplation, contemplation, preparation, action, and maintenance [89]. Sadanshiv et al [66] demonstrated how text messages aligned with these stages, delivering reminders on a predetermined schedule that corresponded to individuals’ progression through the behavior change process.

The social cognitive theory provides a framework for understanding and influencing behaviors through the interplay of personal, environmental, and behavioral factors [90]. A key concept within the social cognitive theory is self-efficacy, which refers to individuals’ belief in their ability to perform behaviors and achieve desired outcomes. Asgary et al [82] structured their messaging around 3 key priorities: knowledge, motivation, and maintenance. When aligned with the social cognitive theory principles, messages addressing disease management, diet, and medication adherence were found to enhance patients’ self-efficacy and confidence, ultimately promoting positive behavioral changes.

Previous medication adherence interventions that used a clearly defined theory tended to achieve better results than those without a theoretical basis [91,92]. Using a single theory to guide message design can simplify the development, analysis, and interpretation of interventions, particularly when the target behavior is clearly defined and the population and setting are specific. This approach allows for a focused exploration of key factors such as beliefs, motivation, or intention. However, it may overlook broader influences such as environmental or social determinants [59]. In contrast, a multitheory approach offers a more comprehensive understanding of the diverse factors influencing medication adherence and provides greater flexibility for designing multicomponent interventions. While this can enhance the richness of the intervention, it also introduces added complexity and requires careful integration to avoid redundancy or conflicting constructs [14,93]. Each behavioral theory contributes unique and validated constructs that can support the development of targeted messages, but overreliance on theory alone may oversimplify the nuanced realities of patient behavior [94]. Human motivation, social context, and environmental influences often do not conform neatly to theoretical models. Therefore, integrating patient feedback alongside theoretical frameworks can help ensure that message design reflects real-world experiences and communication needs [95]. Researchers must strike a balance between theoretical rigor and practical relevance, tailoring their approach to the research goals and intervention context. In exploratory phases, multiple theories may be useful for identifying a broad range of adherence-related factors, while in the development phase, a well-matched single theory or integrated model can guide strategy. During validation, empirical testing, such as structural equation modeling, can help determine which constructs are most predictive in practice. Ultimately, effective messaging depends not only on theoretical soundness but also on contextual relevance and feasibility.

### Tailored or Personalized Messages in Interventions

Growing evidence suggests that, under certain conditions, tailored interventions are more effective in promoting behavior change [96]. Tailored text messages, in particular, have proven to be a valuable tool for improving medication adherence, as they seamlessly integrate into daily routines and can be adapted in real time to meet individual needs and health conditions [97]. Among various tailoring strategies, customization based on individual information is one of the simplest and most widely used approaches. Beyond incorporating basic personal details, such as a patient’s name, birthday, or preferred language, messages can also be personalized based on clinical characteristics, including smoking status or comorbidities, ensuring that the content remains relevant and meaningful to the recipient [75,84]. This level of personalization not only enhances patient engagement but also helps reduce user fatigue from receiving messages that do not align with their personal circumstances [98].

Most users find tailored text message timing and frequency highly beneficial for improving medication adherence. There is a strong demand for the ability to modify these features to better suit individual needs [41]. Allowing patients to adjust reminder messages in accordance with their medication regimen helps enhance adherence while mitigating the potential negative effects of excessive messaging [45,99]. Unlike other tailoring approaches where adjustments are made by the message provider, user-controlled customization of timing and frequency empowers individuals to tailor their messaging experience independently [41]. This emphasis on autonomy in tailored text messaging may significantly enhance engagement with health interventions [100].

Patient-reported assessments offer an effective means of tailoring text messages to improve both patient understanding of medication adherence and their acceptance of the messages themselves [36]. Tailoring can occur at different stages of an intervention. For example, assessments conducted before the intervention can help identify the most relevant and beneficial messages using theory-based tools to uncover key barriers to adherence or by evaluating lifestyle habits and disease status to establish personalized disease management goals [35,36,79]. Meanwhile, assessments conducted during the intervention can track patients’ actual medication use and disease management progress, enabling real-time adjustments—whether to reinforce adherence or to provide reminders for improvement [22,58]. Participants have responded positively to these tailored messages [36]. However, a relatively small proportion of the text messages reviewed in this study incorporated patient-reported assessments for tailoring. This is likely because of the significant resources required, including personnel for assessments and the development of tagging systems to link assessment results with personalized message content [35,36,67].

### User Feedback in Text Messaging Design

Most studies relied on patients’ baseline characteristics at the time of enrollment, while relatively few incorporated changes in patients’ conditions or feedback during or after the intervention period [22,61,71,74]. Integrating weekly feedback via text messages allows researchers to track participants’ medication adherence over the past week, assess the effectiveness of the messages in supporting medication management, and identify the most impactful elements for promoting adherence [22]. In addition, messages can be tailored for subgroups with different disease management goals. For example, Champoux et al [61] demonstrated that weekly or monthly feedback messages provided patients with an overview of their blood pressure management, including comparisons with previous weeks or months, thereby enhancing personal relevance.

Lauffenburger et al [71] combined electronic pill bottles with tailored text messages in their intervention. This approach enabled real-time data collection on the frequency and timing of pill bottle openings, which were then linked to different types of messages. A reinforcement learning algorithm was used to determine which factors should be emphasized in each message, ensuring that the most relevant message was sent to each patient daily. Similarly, Bartlett et al [74] allowed participants to respond to messages with “like” or “dislike” feedback. Messages marked as “liked” were twice as likely to incorporate the same behavioral components in future messages, whereas those marked as “disliked” were half as likely to be repeated. This personalized approach aligned interventions with participants’ habits and preferences, leading to improved effectiveness. Participant feedback provided valuable user-centered insights that enhanced message design, ultimately improving engagement and adherence to both the messages and prescribed medications. However, implementing feedback systems poses challenges, including the increased complexity of managing a larger message bank, reliance on participants’ willingness to provide feedback, and potential privacy concerns owing to the collection of additional personal data.

In summary, the selected text messages demonstrate a range of content development and tailoring strategies designed to align with patients’ specific characteristics and needs. Each approach highlights its feasibility as a viable method for message development. However, the absence of comparative studies makes it unclear which theory-driven designs or tailored approaches yield the best outcomes for improving medication adherence. To develop more effective interventions, it is essential for designers to consider user preferences, the specific characteristics and needs of target patients, and the available resources for message development. Message design should be adapted to fit different situations. When patient background information or personal characteristics are not available in advance, simpler message designs may be more appropriate. However, whenever feasible, incorporating behavioral theory into message development is recommended, especially when it aligns with the goals of the intervention. Ultimately, a balance is needed between the level of message personalization and the overall feasibility of delivering the intervention.

### Strengths and Limitations

This review provides an in-depth analysis of text message design strategies aimed at improving medication adherence. It outlines commonly used theoretical models for message development and tailored approaches, which offer valuable insights for future SMS text messaging interventions. However, several limitations should be acknowledged.

Because of time constraints and limited research capacity, we did not contact study authors or search for related formative publications to retrieve missing information. Consequently, some studies may have been excluded based on insufficient reporting rather than an actual absence of theoretical justification or rationale for message design. Future reviews may benefit from incorporating follow-up strategies to ensure a more comprehensive inclusion of relevant studies.

The study specifically focused on cardiometabolic syndrome, such as hypertension, diabetes, and hyperlipidemia, given their high prevalence and relatively stable treatment regimens. Acute and self-limiting conditions, such as influenza or fractures, typically resolve within a defined period and require minimal long-term intervention, making them less relevant to this review. In addition, this study primarily explored the design of text messages rather than the overall effectiveness of the interventions. Some of the selected studies described tailored message development without reporting outcomes, limiting the ability to evaluate the impact of specific message strategies on adherence. Future studies should evaluate how different text messaging approaches influence psychosocial and clinical outcomes.

Finally, interventions can incorporate multiple communication formats, including text and voice messages, phone calls, or video interactions. While this study focused exclusively on text messages, addressing different health behaviors may require varied approaches. A multifaceted strategy integrating multiple communication channels could offer a more comprehensive solution to overcoming barriers to medication nonadherence.

### Conclusions

The comparison of text message designs offers unique insights beyond integrated quantitative effectiveness. By incorporating behavioral theory and tailored content, text messages can more effectively promote health behaviors. For patients with diabetes, hypertension, and hyperlipidemia on stable treatment, text messages are a practical tool for supporting medication adherence. Personalizing the information can enhance its relevance and strengthen patient engagement while incorporating behavioral theory provides a strong foundation for content design. Together, these strategies promise to promote sustained adherence and encourage positive health behaviors.

## Appendix

Multimedia Appendix 1 Formulating the research question with the SPIDER (sample, phenomenon of interest, design, evaluation, research type) framework.

Multimedia Appendix 2 Process of conducting a literature search on the PubMed and Scopus databases.

Multimedia Appendix 3 PRISMA-ScR (Preferred Reporting Items for Systematic Reviews and Meta-Analyses Extension for Scoping Reviews) checklist.

## Abbreviations

COM-B: capability, motivation, opportunity, behavior
PRISMA-ScR: Preferred Reporting Items for Systematic Reviews and Meta-Analyses Extension for Scoping Reviews
SPIDER: sample, phenomenon of interest, design, evaluation, research type
