# Supplementary material for: Key Elements and Theoretical Foundations for the Design and Delivery of Text Messages to Boost Medication Adherence in Patients With Diabetes, Hypertension, and Hyperlipidemia: Scoping Review
Source: J Med Internet Res. 2025 Jul 21;27:e71982. doi: 10.2196/71982 (PMC12322613; doi:10.2196/71982)
Supplement: Multimedia Appendix 1 [file jmir_v27i1e71982_app1.docx]

**Appendix 1. Formulating the research question with the SPIDER framework**

| **SPIDER tool^a^** | **Definition** | **Search Terms/Strategies** |
| --- | --- | --- |
| S - Sample | cardiometabolic syndrome | “diabetes” OR “hypertension” OR “hyperlipidemia” |
| PI - Phenomenon of Interest | text message | “message” OR “text” OR “text message” |
| D - Design | digital device or application | “app” OR “application” OR “device” OR “digital” |
| E - Evaluation | medication adherence | “medical adherence” OR “medication adherence” |
| R - Research Type | all types of research excluding review | Not“review [title]” |

^a^ (S) AND (PI) AND (D) AND (E) AND (R)
