# Supplementary material for: Key Elements and Theoretical Foundations for the Design and Delivery of Text Messages to Boost Medication Adherence in Patients With Diabetes, Hypertension, and Hyperlipidemia: Scoping Review
Source: J Med Internet Res. 2025 Jul 21;27:e71982. doi: 10.2196/71982 (PMC12322613; doi:10.2196/71982)
Supplement: Multimedia Appendix 2 [file jmir_v27i1e71982_app2.docx]

**Appendix 2. Process of conducting a literature search on PubMed and Scopus**

**Search term for PubMed:**

(((((diabetes) OR (hypertension) OR (hyperlipidemia)) AND ((message) OR (text) OR (text message))) AND ((app) OR (application) OR (digital) OR (device) OR (mobile))) AND ((medical adherence) OR (medication adherence))) NOT (review[Title])

**Search term for Scopus:**

(TITLE-ABS-KEY ((diabetes) OR (hypertension) OR (hyperlipidemia))) AND (TITLE-ABS-KEY ((message) OR (text) OR (text AND message))) AND (TITLE-ABS-KEY ((app) OR (application) OR (digital) OR (device))) AND (TITLE-ABS-KEY ((medical AND adherence) OR (medication AND adherence))) AND NOT (TITLE ((review)))
